# AgNPs treatment reduces time recovery and increases bacterial sensitivity to antibiotics in cow´s purulent catarrhal endometritis. A translational study

**DOI:** 10.1371/journal.pone.0335305

**Published:** 2025-10-29

**Authors:** Alejandro Huerta-Saquero, Ekaterina Nefedova, Nikolay N. Shkil, Alexey Pestryakov, Nina Bogdanchikova

**Affiliations:** 1 Centro de Nanociencias y Nanotecnología, Universidad Nacional Autónoma de México, Ensenada, Baja California, México; 2 Siberian Federal Scientific Centre of Agro-BioTechnologies of the Russian Academy of Sciences, Novosibirsk, Russia; 3 Research School of Chemistry and Applied Biomedical Sciences, Tomsk Polytechnic University, Tomsk, Russia; Beni Suef University Faculty of Veterinary Medicine, EGYPT

## Abstract

Cow purulent catarrhal endometritis (PCE) is a common reproductive disorder in dairy cattle caused by bacterial infections. PCE impacts fertility, milk production, and animal health. Therapeutic approaches include systemic or intrauterine antibiotics. Unfortunately, the overuse of antibiotics in treating PCE drives the emergence of antibiotic-resistant bacteria, making infections difficult to treat and increasing food safety concerns due to antibiotic residues remaining in milk and meat products, posing health risks to consumers. Antimicrobial nanomaterials, particularly silver nanoparticles (AgNPs), provide an efficient alternative to combat multi-resistant bacteria, and the synergistic activity of AgNPs and antibiotics has been well documented, making the treatments of bacterial infections more efficient. Here, a comparative study is shown applying Argovit-C (AgNPs) and Enrocide as therapeutics for treating PCE in cattle. Intrauterine application of Argovit-C reduces the recovery time of cattle in comparison with Enrocide treatment as well as increases the sensitivity to antibiotics of *Escherichia coli* isolates from cervical canal secretion samples of diseased cattle. The increased sensitivity was found to 24 antibiotics, including aminoglycosides, fluoroquinolones, tetracyclines, penicillins, cephalosporins, macrolides, polymyxin, rifampicin, and chloramphenicol. The increased sensitivity was much higher for those bacteria that did not show an active efflux effect. Furthermore, Argovit-C reduced the acquisition of *blaDHA* and *blaGES* resistance genes in *E. coli*, as well as the number of bacterial isolates without efflux effect. Overall, this translational study performed in 300 cows demonstrates the ability of Argovit-C AgNPs to combat bacterial infections, favoring an increase in bacterial susceptibility to antibiotics and reducing their ability to acquire antibiotic multi-resistant genes.

## 1. Introduction

Cow purulent catarrhal endometritis (PCE) is a common reproductive disorder in dairy cattle, characterized by inflammation of the endometrium, the inner lining of the uterus, accompanied by purulent (pus-containing) discharge. This condition is primarily observed postpartum and can significantly impact fertility, milk production, and overall herd health. The etiology of PCE is multifactorial, involving bacterial infections, particularly by pathogens such as *Escherichia coli*, *Trueperella pyogenes*, and *Fusobacterium necrophorum*, often in conjunction with other opportunistic bacteria, such as *Salmonella enteritidis* and *Pseudomonas aeruginosa* [1,2].

The pathogenesis of PCE involves invading these pathogenic bacteria into the uterus, usually facilitated by predisposing factors like dystocia, retained placenta, and poor uterine involution. These conditions create an environment conducive to bacterial growth and subsequent inflammation. The clinical manifestations of PCE include abnormal uterine discharge, systemic signs of infection like fever and anorexia, and, in severe cases, toxemia. The diagnosis is typically based on clinical examination, palpation, and the presence of a foul-smelling uterine discharge [3,4]. Preventative strategies emphasize proper calving management, hygiene, and nutrition to enhance uterine health and reduce the risk of infections. Early detection and treatment are crucial for minimizing the adverse effects on the affected animals’ reproductive performance and overall productivity [5]. Effective management of PCE involves a combination of therapeutic approaches, including systemic or intrauterine antibiotics treatments, anti-inflammatory drugs, and supportive care. One of the first-line treatments for PCE is the intrauterine administration of Enrocide (enrofloxacin, a broad-spectrum bactericidal antibiotic from the fluoroquinolone family), among other broad-spectrum antibiotics [6]. The risks associated with the overuse of antibiotics in treating PCE in cows include the emergence of antibiotic-resistant bacteria, making future infections more difficult to treat, reduced efficacy of antibiotics, disruption of cow’s microbiome, increasing susceptibility to other infections, and food safety concerns due antibiotic residues can remain in milk and meat products, posing health risks to consumers [7,8]. Given this scenario, there is an urgent need to use new antimicrobial agents to mitigate infections caused by bacteria with multiple antibiotic resistance and, concomitantly, reduce their use to avoid contamination of livestock products towards the final consumer. In this sense, antimicrobial nanomaterials, particularly silver nanoparticles (AgNPs), provide an efficient alternative to combat multi-resistant bacteria. Moreover, the synergistic activity of AgNPs and antibiotics has been well documented, making the treatments of bacterial infections more efficient [9–11]. We have reported the efficacy of Argovit-C as an antimicrobial agent that kills bacteria, yeasts, fungi, unicellular eukaryotes, animal and cancer cells in culture, and even its interference in the infectious process of viruses, which prevents their internalization into host cells, and therefore, prevents their proliferation [12]. It has also been shown that AgNPs cause a synergistic antimicrobial effect in combination with conventional antibiotics, mainly those whose mechanism of action requires entry into the cell [11]. We previously reported large-scale translational studies showing that AgNPs (Argovit-C) treatments increased the susceptibility of *Staphylococcus aureus* and *Streptococcus dysgalactiae* to antibiotics [13,14]. as well as in mastitis treatments in cows, where *E. coli* showed reduced resistance to antibiotics due to Argovit-C treatment [15]. This synergistic activity between antibiotics and AgNPs reduces treatment time and the disappearance of the infection symptoms. Among the mechanisms of action, it has been proposed that AgNPs alter efflux pump activity, by decreasing the efflux pump gene expression and, thus, by reducing the functional efflux pump proteins [16]. The influence of AgNPs on the efflux pump proteins, named “efflux effect” was documented in *P. aeruginosa*, *E. coli*, *Acinetobacter baumannii*, *Klebsiella pneumonie*, *Burkholderia pseudomallei*, and *Enterobacter cloacae*, among others [17–22]. These effects showed the capability of Argovit-C AgNPs to reduce the antibiotics-efflux effect, favoring an increase in bacterial susceptibility to antibiotics.

This translational study aimed to compare the efficacy of intrauterine application of Argovit-C or Enrocide as therapeutics for treating PCE in cattle, determining the recovery time for each of them and the *in vitro* sensitivity of *E. coli* isolates to different antibiotics, and also, determining the *E. coli* ability to acquire the *blaDHA* and *blaGES* resistance genes.

## 2. Results

### 2.1. *In vivo* studies

#### 2.1.1. Sample collection and bacterial isolation.

The vaginal microbiome of cattle with PCE suffers from dysbiosis due to the proliferation of pathogenic organisms. It was essential to characterize first the presence of pathogenic bacteria causing the disease, for which cervical canal samples were obtained from the 300 sick cattle before the treatments. We found that mainly *Salmonella enteritidis*, *Escherichia coli*, and *Pseudomonas aeruginosa* were isolated at 56.6, 40 and 5%, respectively (Table 1). The three bacteria have been widely characterized, making it common to find multi-antibiotic-resistant varieties. This study focused on the further isolation and characterization of *E. coli*.

**Table 1:** Microbiological study of the cervical canal mucus secretion from 300 cows with purulent catarrhal endometritis.

Microorganisms	Endometritis	
	Number of isolates	%
*S. enteritidis*	170	56,6
*E. coli*	120	40
*P. aeruginosa*	10	5

#### 2.1.2 Intrauterine infusion.

Three hundred cattle diagnosed with PCE were divided into two groups, which were treated with Argovit-C or with Enrocide. Argovit-C or Enrocide was intrauterine administered daily, and disease symptoms were checked. On average, cattle administered with Argovit-C showed a total recovery time of 7.8 days, while those treated with Enrocide required a longer treatment time until recovery, with an average time of 12.6 days (Table 2).

**Table 2:** Recovery time of cows with postpartum PCE after treatments.

Treatment groups	Recovery time (in days)
Argovit-C	7.8 ± 0.1
Enrocide	12.6 ± 0.3

The values show the average recovery time of 300 cows expressed in days ± SD.

## 3. *In vitro* studies

### 3.1. *In vitro* antibiotic sensitivity

After identification of the pathogenic bacteria causing dysbiosis in cattle, *E. coli* was isolated before and after each treatment. *E. coli* isolates were subjected to a sensitivity test for multiple antibiotics from different families possessing various mechanisms of action. The groups of antibiotics tested included aminoglycosides (5), fluoroquinolones (4), tetracyclines (3), penicillin (4), cephalosporins (2), macrolides (2) and other groups (4). The total number of antibiotics tested was 24, as shown in S2 and S3 Tables. The same bacterial isolates were subjected to the efflux effect test to characterize those isolates with active efflux and those isolates that did not present it and to evaluate whether this characteristic influence resistance to the different antibiotics tested before and after each treatment.

#### 3.1.1. Enrocide treatment.

The first-line treatment for PCE is Enrocide. This drug was administered intrauterine, and the disappearance of disease symptoms was monitored. After this treatment, *E. coli* was isolated from cows and exposed to sensitivity tests to 24 antibiotics from different groups. Bacterial isolates from cervical canal samples were divided into those with positive and negative “efflux effect”. The results of antibiotic sensitivity are shown in S2 Table.

**Fig 1:**
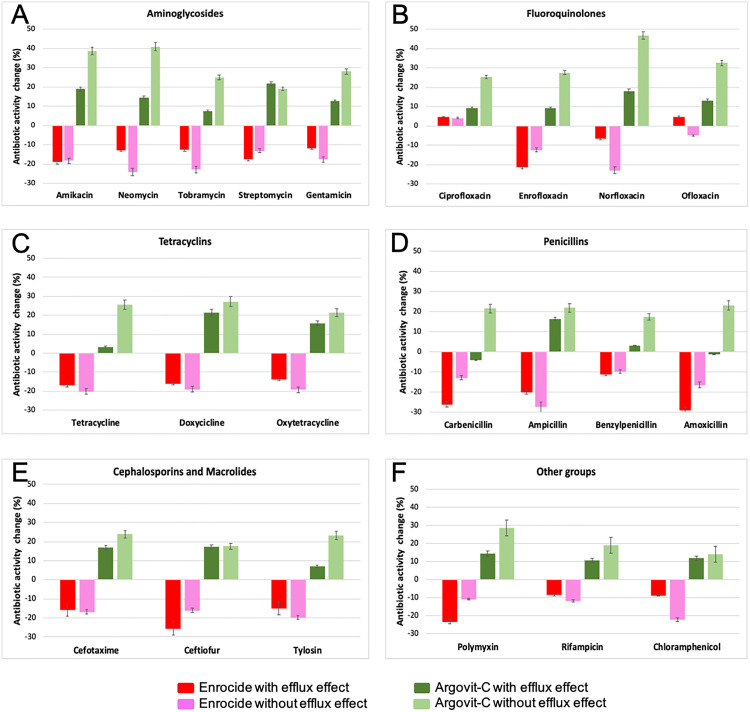
Sensitivity of *E. coli* isolates to antibiotics. Bars represent the percentage of the increment or decrement of antibiotic activity on *E. coli* isolates (n) from cervical canal samples of cows with PCE after treatment with Enrocide (red and pink bars) or Argovit-C (dark green and light green bars). Bacteria were divided according to the presence of the efflux effect (red and dark green bars) or the absence of the efflux effect (pink and light green bars).

Sensitivity to the aminoglycoside’s amikacin, neomycin, tobramycin, streptomycin, and gentamicin was reduced by 11.7 to 19% in bacteria with an efflux effect (Fig 1A, red bars) and by 13.2 to 24% in those bacteria that did not show active efflux (Fig 1A, pink bars). The sensitivity of *E. coli* with efflux effect to the fluoroquinolone, ciprofloxacin and ofloxacin increased by 4.6 and 4.8%, respectively. In comparison, it was reduced for enrofloxacin and norfloxacin by 21.2 and 6.7%, respectively (Fig 1B, red bars). *E. coli* without efflux effect reduced sensitivity to enrofloxacin, norfloxacin, and ofloxacin (12.4, 23 and 5%, respectively), increasing only for ciprofloxacin by 4.1% (Fig 1B, pink bars).

*E. coli* also showed lower sensitivity to tetracyclines (tetracycline, doxycycline, and oxytetracycline) with efflux effect (Fig 1C, red bars) or without efflux effect (Fig 1C, pink bars), ranging from 13.7 to 20.1% reduction in sensitivity to this type of antibiotics. Similarly, a consistent reduction in sensitivity was found for the tested penicillins (carbenicillin, ampicillin, benzylpenicillin, and amoxicillin), with and without efflux effect, in a range between 9.6 and 29% (Fig 1D, red and pink bars, respectively). For the cephalosporins cefotaxime and ceftiofur, as well as the macrolide tylosin, sensitivity dropped between 14.9 and 25.5% in *E. coli* with efflux effect and between 16.1 and 19.7 in those without efflux (Fig 1E, red and pink bars, respectively). All *E. coli* isolates were resistant to erythromycin and lincomycin.

Finally, Enrocide treatment also negatively impacted the effectiveness of the antibiotics polymyxin, rifampicin, and chloramphenicol by reducing the sensitivity of *E. coli*, with and without efflux effect (Fig 1F, red and pink bars, respectively).

#### 3.1.2. Argovit-C treatment.

**Fig 2:**
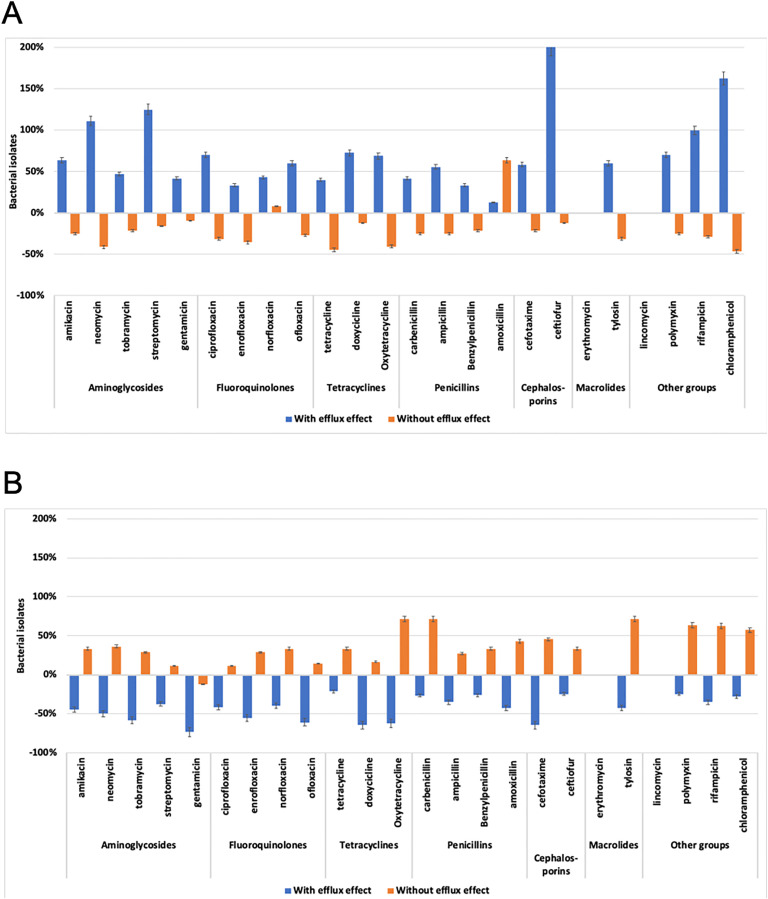
Bacterial isolates with efflux effect depend on Enrocide or Argovit-C treatment. Percentage of change of the number of *E. coli* isolates with (blue bars) and without (orange bars) efflux effect. **A)** After Enrocide or **B)** Argovit-C treatments.

Unlike Enrocide treatment, Argovit-C treatment of cows with PCE showed a spectacular increment of sensitivity of *E. coli* isolates to antibiotics of all groups tested (S3 Table, Fig 2). The sensitivity of *E. coli* to the aminoglycoside’s amikacin, neomycin, tobramycin, streptomycin, and gentamicin showed an increment of 7.6 to 21.7% in bacteria with an efflux effect (Fig 1A, dark green bars), and by 19.1 to 40.9% in those bacteria that did not show active efflux (Fig 1A, light green bars). The sensitivity of *E. coli* with efflux effect to the fluoroquinolones increased by 9.1 to 17.9%. In contrast, the increment was more significant in *E. coli* without efflux effect, ranging from 25.4 to 46.9% (Fig 1B, dark green and light green bars, respectively). *E. coli* also showed an increased sensitivity to tetracyclines (tetracycline, doxycycline, and oxytetracycline) with efflux effect (Fig 1C, dark green bars) or without efflux effect (Fig 1C, light green bars), ranging from 3.4 to 21.4% increase in sensitivity to this type of antibiotics. On the other hand, a slight decrement in sensitivity was found for carbenicillin and amoxicillin in bacteria with efflux effect. In contrast, a more significant sensitivity increment was found for all penicillin’s tested in bacteria without efflux effect and even ampicillin and benzylpenicillin in bacteria with efflux effect (Fig 1D, dark green and light green bars, respectively). Cephalosporin results were consistent in increasing the sensitivity of bacteria regardless of the efflux effect, ranging from 16.7 to 23.8% of the increment (Fig 1E, dark green and light green bars, respectively). The treatment also increments the effectiveness of the macrolide tylosin, increasing sensitivity by 7.1 and 23.1% in bacteria with and without efflux effect, respectively (Fig 1E, dark green and light green bars). Finally, treatment with Argovit-C also increments the effectiveness of the antibiotics polymyxin, rifampicin, and chloramphenicol on *E. coli*, with and without efflux effect (Fig 1F, dark green and light green bars, respectively).

### 3.2. Efflux effect after Enrocide treatment

*E. coli* bacterial isolates obtained from samples of cows with PCE were evaluated to determine if they had active efflux or no efflux effect, using the Eugonic agar-ethidium bromide technique, as described in materials and methods. After treatment with Enrocide, the number of bacterial isolates with efflux effect showed an increase ranging from 125 to 42% in bacteria exposed to the aminoglycoside group, from 70 to 33% for fluoroquinolones, from 73 to 40% for tetracyclines; from 56 to 13% for penicillins; from 200 and 58% for cephalosporins; 60% for tylosin and 163, 100 and 70% increase in isolates with efflux effect in bacteria exposed to chloramphenicol, rifampicin and polymyxin, respectively. (Fig 2A, blue bars). Likewise, Enrocide treatment reduced the percentage of bacterial isolates without efflux effect exposed to most of the antibiotics analyzed (except norfloxacin and amoxicillin) (Fig 2A, orange bars). On average, the number of bacterial isolates with the efflux effect after Enrocide treatment increased by 71%, whereas the number of isolates without the efflux effect decreased by an average of 21%. There was one exception (norfloxacin) which increase isolated without efflux effect by 8% (Table 3).

**Table 3:** Change in the number of *E. coli* isolates with and without efflux effect after treatment with Enrocide or Argovit-C.

Treatment	Isolates with the Efflux Effect		Isolates without the Efflux Effect	
	Number of antibiotics	Average change %	Number of antibiotics	Average change %
Enrocide	22	+71	22	−21
Argovit-C	22	−44	22	+37

### 3.3. Efflux effect after Argovit-C treatment

In contrast to Enrocide treatment, *E. coli* bacterial isolates obtained from cervical canal samples of cows with PCE treated with Argovit-C showed a decrease in those with active efflux, ranging from 74 to 38% in bacteria exposed to aminoglycosides, from 61 to 42% for fluoroquinolones, from 65 to 21% for tetracyclines, from 43 to 26% for penicillins, from 65 and 25% decrease for isolates exposed to cefotaxime and ceftiofur (cephalosporins), from 43% for tylosin, and a decrease in isolates with efflux effect exposed to polymyxin, rifampicin and chloramphenicol (25, 35 and 28% respectively; Fig 2B, blue bars). Likewise, a discrete increase was observed in the number of isolates without efflux effect exposed to antibiotics in all groups analyzed, except isolates exposed to gentamicin (Fig 2B, orange bars). On average, the number of bacterial isolates with the efflux effect after Argovit-C treatment decreased by 44%, whereas those isolates without the efflux effect increased an average by 37%. Gentamicin treatment decreased efflux effect by 13% of bacterial isolates (Table 3).

### 3.4. *bla* genes disemination after Enrocide or Argovit-C treatment

A central problem in antibiotic treatment for infections caused by bacteria is the selection of bacteria resistant to conventional antibiotics and the dissemination of this resistance through horizontal gene transfer events, which gives rise to multi-resistance mediated by the acquisition of resistance genes. In this regard, we were interested in evaluating the presence of resistance genes in *E. coli* isolates from cows with endometritis, based mainly on β-lactamase genes (*blaGES* and *blaDHA*, conferring resistance to carbapenems and cephalosporins/penicillins, respectively).

Table 4 shows the antibiotic resistance genes of *E. coli* isolated before and after treatment with Enrocide from cows with purulent catarrhal endometritis. It can be observed that the presence of *blaGES* in *E. coli* samples isolated from cervical canal mucus was detected in 12.5% of isolates obtained prior to treatment with Enrocide. In contrast, after treatment with Enrocide, the *blaGES* gene increased to 37.5%. On the other hand, the presence of the *blaDHA* resistance gene, which confers resistance to carbapenems, cephalosporins, and penicillins, was detected in 8.3% of *E. coli* samples isolated from cervical canal mucus before treatment of PCE of cows with the drug Enrocide. In contrast, after treatment, the presence of the *blaDHA* gene was found in 29.2% of isolates.

**Table 4:** Antibiotic resistance genes of *E. coli* isolates from cervical canal samples of cows with PCE, before and after Enrocide or Argovit-C treatments.

Treatment	Antibiotics	Gene	Contribution of number of isolates carrying the specific gene in total number of isolates (%)		
			Before treatment	After treatment	Change after treatment
*Enrocide*	Carbapenems	*blaGES*	12.5	37.5	+25
	Cephalosporins Penicillins	*blaDHA*	8.3	29.2	+20.9
*Argovit-C*	Carbapenems	*blaGES*	12.5	8.3	−4.2
	Cephalosporins Penicillins	*blaDHA*	8.3	4.2	−4.1

On the other hand, *blaGES* and *blaDHA* genes were detected in *E. coli* isolates from cervical canal mucus samples of cows with endometritis prior to treatment with Argovit-C. It was found that 12.5 and 8.3% of the samples were positive for the *blaGES* and *blaDHA* genes, respectively. After treatment with Argovit-C, the *E. coli* isolates positive for the presence of the *blaGES* and *blaDHA* genes were reduced to 8.3 and 4.2% of the samples, respectively (Table 4).

## 4. Discussion

The treatment of infectious diseases represents a challenge for the health sector of all countries, generating significant human and economic losses. Infectious diseases in farm animals and animals for human consumption cause several serious problems, such as high mortality/morbidity, a decrease in the production of resources (meat, milk, and related products for human consumption), a decrease in reproduction, economic losses related to treatment, among others. As if that were not enough, using antibiotics to combat infectious diseases has generated the development of bacteria with multi-resistance to antibiotics available on the market, aggravating the situation and reducing the chances of successful treatment. In this context, the search for new antimicrobials has allowed the development and characterization of antimicrobial nanomaterials, mainly metallic nanoparticles, highlighting those of silver. Bovine endometritis is caused by the proliferation of different pathogenic bacteria, which generate dysbiosis in the uterine microbiome and cause severe health damage to livestock, with corresponding million-dollar losses. In this work, we report an alternative treatment for bovine endometritis, using Argovit-C as an antimicrobial agent, which are AgNPs that stand out from others for their antimicrobial efficiency, stability, and effectiveness in the treatment of various infectious diseases in animals, plants, and even humans [23–25]. Their effectiveness has been documented for treating canine distemper, bovine mastitis, and even skin wounds, as composite in wound dressings [13–15,26,27].

The antimicrobial effect of AgNPs has been widely documented. AgNPs main effect on cells is at the membrane level, causing membrane depolarization and affecting its permeability, which leads to the uncontrolled entry of metabolites (for example, antibiotics), causing cell lysis [11]. Another important mechanism is the alteration of outer membrane proteins (OMPs) function, which are responsible for the efflux of toxic metabolites in bacteria [16]. It has been documented that AgNPs provoke efflux systems dysfunction and, thus, increases bacterial susceptibility to antimicrobials [13–15].

During PCE, cattle suffer infections with *E. coli, S. enteritidis, and P. aeruginosa*. A high percentage of the 300 cows analyzed in this study showed the presence of *E. coli* (40%). Treatment with Enrocide and Argovit-C had significant differences regarding the recovery time of cattle, with Argovit-C being 38% more efficient than Enrocide reducing recovery time from 12.6 to 7.8 days in average, and mainly in the reduction of *E. coli* resistance to the antibiotics from different groups. The antimicrobial capacity of AgNPs and their synergy with different antibiotics have been well documented [28–31]. However, most of the reports include *in vitro* experimentation. The translational study presented here demonstrated the efficiency of using AgNPs in PCE *in vivo*, revealing an increase of the sensitivity of *E. coli* to 24 antibiotics from at least six groups. This increase in sensitivity is mainly observed in those isolated bacteria that presented an altered or even absent efflux effect. It is worth mentioning that treatment with Enrocide, containing a fluoroquinolone caused *E. coli* to increase its sensitivity to two antibiotics of this group only (ciprofloxacin and ofloxacin). However, these increases were very small (<5%). Another significant result is that Argovit-C treatment reduces the number of *E. coli* isolates with an efflux effect, which at least partially explains the increase in the sensitivity of the bacteria to antibiotics since it alters the efflux pumps, one of the main detoxification mechanisms that participate in the mechanisms of resistance to antibiotics. It is important to distinguish that our work did not evaluate the already well-known antimicrobial synergism between AgNPs and antibiotics but rather revealed a sensitization mechanism of bacteria exposed to AgNPs that reduced their resistance to the antibiotics used. It is important to mention that the results obtained with Argovit-C for combating pathogenic microorganisms causing endometritis are similar to those obtained for treating microorganisms causing mastitis in cows, such as *E. coli, S. aureus,* and *S. dysgalatiae* [13–15]. In particular, for *E. coli* infections, in both PCE and mastitis, there was an increase in sensitivity to antibiotics of all families tested (S1 Fig and S4 Table). For instance, in PCE *E. coli* isolates with active efflux effect, an increase in sensitivity ranges from 3.6 to 53.6% for penicillins and macrolides, respectively, whereas in mastitis range from 11.5 to 30.6 for macrolides and aminoglycosides, respectively. In the case of *E. coli* isolates without efflux effect, the increase in sensitivity ranges from 20.3 to 61.1 for cephalosporins and macrolides, in the case of PCE; whereas, for mastitis, it ranges from 15.4 to 26.8 for penicillins and aminoglycosides, respectively (S5 Table). Also, in both different infections, there is a reduction in the number of isolates with efflux effect [15]. So, the effects described above were observed for two cattle diseases.

Using conventional antibiotics to treat infections has resulted in the selection of multi-resistant bacteria, which can transfer resistant genes to other bacteria through horizontal gene transfer events. Antibiotic resistance of microorganisms of the Enterobacteriaceae family is associated with the presence of *GES* genes encoding extended-spectrum beta-lactamase (ESBL) enzymes 2be and 2f, belonging to the molecular class A, capable of hydrolyzing penicillins, cephalosporins, and monobactams. The class A carbapenemase encoded by *blaGES* is either plasmid-borne or located on the chromosome of the host. The GES family enzymes comprise four out of nine members with enzymatic activity against carbapenems [32].

On the other hand, the presence of the AmpC-β lactamase *blaDHA* gene mediates resistance to penicillins and cephalosporins, while its overexpression confers resistance to broad-spectrum cephalosporins, including ceftriaxone, cefotaxime, and ceftazidime [33,34]. In this sense, the presence of genes that confer resistance to β-lactams in *E. coli* isolates obtained from cervical canal mucus samples of cattle with PCE was determined. We observed that treatment with Argovit-C significantly reduced the percentage of *blaGES* and *blaDHA* of the isolated bacteria (from 12.5 to 8.3% and from 8.3 to 4.2%, respectively), unlike treatment with Enrocide, where an increase in the percentage of bacteria where these genes were detected. This finding is significant because it indicates that the treatment of bacterial infections, especially those provoked by bacteria with multi-resistance to antibiotics, can include AgNPs such as Argovit-C as an initial treatment before first-line antibiotics use to reduce recovery time, prevent antibiotic-resistance mechanisms, and more importantly, reduce the bacterial capacity for the dissemination of antibiotic resistance genes.

All of the above strongly suggests that treatment with Argovit-C for different infectious diseases will increase bacterial sensitivity to antibiotics, preventing antibiotic-resistance mechanisms and reducing recovery time, regardless of the area of infection and the causative microorganism.

## 5. Conclusions

This translational study demonstrates the increased sensitivity to antibiotics in *E. coli* caused by exposure to Argovit C (AgNPs), which is described for the first time in bovine endometritis.

Using Argovit-C AgNPs for treating veterinary infections such as PCE in cattle reduces by 38% the recovery time of cows and increases the sensitivity to antibiotics of pathogenic bacteria, ranging from 3.6 to 61.6% depending on antibiotic type. Argovit-C treatment interferes with antibiotic-resistance mechanisms such as efflux pumps by reducing active efflux and reduces the dissemination of antibiotic resistance genes in bacteria. Our findings open an avenue for combating bacterial multi-resistance of pathogenic microorganisms to antibiotics, regardless of infectious disease and causative microorganism.

## 6. Methods

This work was carried out on breeding farms with 300 cows suffering from PCE. The diagnosis was based on clinical symptoms. The evaluation for therapeutic effectiveness was: the disappearance of clinical signs of uterine inflammation, improvement in general condition, and recovery fertilization ability. The cows were divided into two equal groups of 150 animals each. The first group included cows that received Enrocide (Agrofarm Research and Production Enterprise CJSC, Russia) and Uteroton (Nita-Pharm LLC, Russia). The second group included 150 cows treated with AgNPs Argovit-C TM (Research and Production Center “Vector-Vita,” Novosibirsk, Russia) and Uteroton. Cervical canal mucus samples from both groups were analyzed before and after their intrauterine treatment (after complete recovery) with these drugs. Samples taken before treatments were considered as a reference (control).

### 6.1. Sampling

Samples of cervical canal mucus secretions were taken from cows with PCE before and after treatment with Enrocide or Argovit-C in the conditions of livestock farms in the Novosibirsk region (Russia) during 2018–2021. Cervical canal secretion was collected using a sterile cotton swab with a gauze swab after thoroughly cleaning the external integument around the vulva and root of the tail, finishing it with a swab moistened with 70% ethyl alcohol rectifier. The test tube with cervical canal secretion was covered with a cotton-gauze plug. Samples were stored at 8–10°C until testing. Within 3–4 hours, samples were delivered for testing.

### 6.2. Treatment formulations

Enrocide, Uteroton, and Argovit-C were used in this study. Enrocide is an antibacterial drug for intrauterine insertion in the form of a clear, pale green liquid color containing enrofloxacin 0.4 g as an active ingredient and as excipients: dimethyl sulfoxide 30 g, sodium hydroxide up to pH 10.5–12.5, and distilled water up to 100 ml. Cows were intrauterine injected with Enrocide 100 ml once every 48 hours until clinical recovery. Uteroton is a medicinal product for intramuscular administration in the form of a colorless liquid containing 5 mg of anapriline (propranolol hydrochloride), as well as 2 mg sodium metabisulfite, 5 mg chlorethone, citric acid to pH 3.5 ± 0.5 and distilled water to 1 ml. All cows were injected intramuscularly with 10 ml Uteroton three times with an interval of 24 hours. Argovit-C is a veterinary drug provided by Dr. Vasily Burmistrov. Applicable for therapeutic and preventive purposes for gastrointestinal diseases in calves, Argovit-C is a stable aqueous suspension of silver nanoparticles (AgNPs) with a concentration of 200 mg/ml (20% weight/volume). The concentration of metallic silver is 12 mg/ml (1.2 wt.%), and the size of silver nanoparticles is 5–20 nm with an average diameter of 15 nm. Argovit-C AgNPs are stabilized with polyvinylpyrrolidone and hydrolysate collagen. 100 ml of a 20-fold dilution of Argovit-C was administered intrauterine in animals of the experimental group with purulent-catarrhal endometritis (n = 150) (1.2 mg metallic Ag/ml final concentration) once every 48 hours until complete recovery.

### 6.3. Isolation and Identification of *E. coli* in cervical canal samples

*E. coli* was isolated from cow cervical canal secretions before and after drug treatments with Enrocide or Argovit-C. Bacterial identification isolated from cervical microbiota was performed by morphological and biochemical properties according to generally accepted methods “Berge’s Guide to Determinative Bacteriology,” 2000 [35].

### 6.4. Antimicrobial susceptibility and efflux testing

The antimicrobial sensitivity test was performed using a Gram-negative infection test kit (LLC NPO Diagnostic Systems, Nizhny Novgorod) following the criteria of the European Committee for antimicrobial susceptibility testing (EUCAST, 2018) [36].

The study of the efflux effect consisted of sowing an *E. coli* culture on Eugonic agar with ethidium bromide (1 mg/l). After 24 hours of cultivation, cultures were detected using a transilluminator. Without the efflux effect, ethidium bromide penetrated the bacterial cell, and its complex with DNA glowed in ultraviolet rays. In contrast, the active efflux effect ensured the absence of fluorescence. Studies on sensitivity and efflux effect on *E. coli* isolated from the cervical canal mucus secretion of cows with PCE were carried out for 24 antibiotics, belonging to 7 groups: 1. Aminoglycosides (amikacin, neomycin, tobramycin, streptomycin, gentamicin), 2. Fluoroquinolones (norfloxacin, enrofloxacin, ciprofloxacin, ofloxacin), 3. Tetracyclines (tetracycline, doxycycline, oxytetracycline), 4. Penicillins (carbecillin, ampicillin, benzylpenicillin, amoxicillin), 5. Cephalosporins (ceftiofur, cefotaxime), 6. Macrolides (erythromycin, tylosin), 7. Others (lincomycin, polymyxin, rifampicin, chloramphenicol). Sensitivity and efflux studies were conducted before and after treatment with drugs of different pharmacological groups (Argovit-C and Enrocide).

### 6.5. PCR Detection of antibiotic resistance genes in *E. coli* from cervical canal mucus isolates of cows with PCE

The detection of genes that confer resistance to carbapenems (*blaGES* gene, encoding a carbapenemase) [37], cephalosporins and penicillins (*blaDHA*, encoding β-lactamase) [38] was carried out by end-point PCR, of total DNA of *E. coli* isolates from cervical canal mucus samples of cows with purulent catarrhal endometritis, before and after treatments with Enrocide and Argovit-C, respectively. The amplification conditions were as follows: 95°C - 1 min 30 s; and 40 cycles of 95°C – 15 s, 60°C – 30 s, 72°C – 40 s. The sequence of the oligonucleotides used is shown in S1 Table.

### 6.6. Ethics statement

The experimental protocols were approved by Ethical Committee of Federal State Budgetary Institution of Siberian Federal Scientific Center for Agrobiotechnologies of Russian Academy of Sciences (decision No. 00017 from 10th February 2017).

### 6.7. Statistical analysis

Average and standard deviation calculations statistically processed the results. Results were corrected, systematized, and visualized using GraphPad Software 9.0, San Diego, CA, USA.

Information files.

## Supporting information

S1 Data Efflux effect. (XLSX)

S2 Data Antibiotic sensitivity. (ZIP)

S1 Fig Antibiotic sensitivity changes of *E. coli* isolates from Purulent catarrhal endometritis and mastitis. (DOCX)

S1 Table Primer sequences for detection of resistance genes. (DOCX)

S2 Table Antibiotic sensitivity test of *E. coli* isolates from cows’ PCE before and after treatment with Enrocide. (DOCX)

S3 Table Antibiotic sensitivity test of *E. coli* isolates from cow´s PCE before and after treatment with Argovit-C. (DOCX)

S4 Table Comparison of antibiotic sensitivity change of *E. coli* isolates from Purulent Catharral Endometritis (PCE) and mastitis after Argovit-C treatments. (DOCX)

S5 Table Average of antibiotic sensitivity change of *E. coli* isolates from PCE and mastitis after Argovit-C treatments. (DOCX)
