# Supplementary material for: AgNPs treatment reduces time recovery and increases bacterial sensitivity to antibiotics in cow´s purulent catarrhal endometritis. A translational study
Source: PLoS One. 2025 Oct 29;20(10):e0335305. doi: 10.1371/journal.pone.0335305 (PMC12571309; doi:10.1371/journal.pone.0335305)
Supplement: S1 Fig — (DOCX) [file pone.0335305.s001.docx]

**Supplementary Fig 1.** Antibiotic sensitivity changes of *E. coli* isolates from Purulent catarrhal endometritis and mastitis.


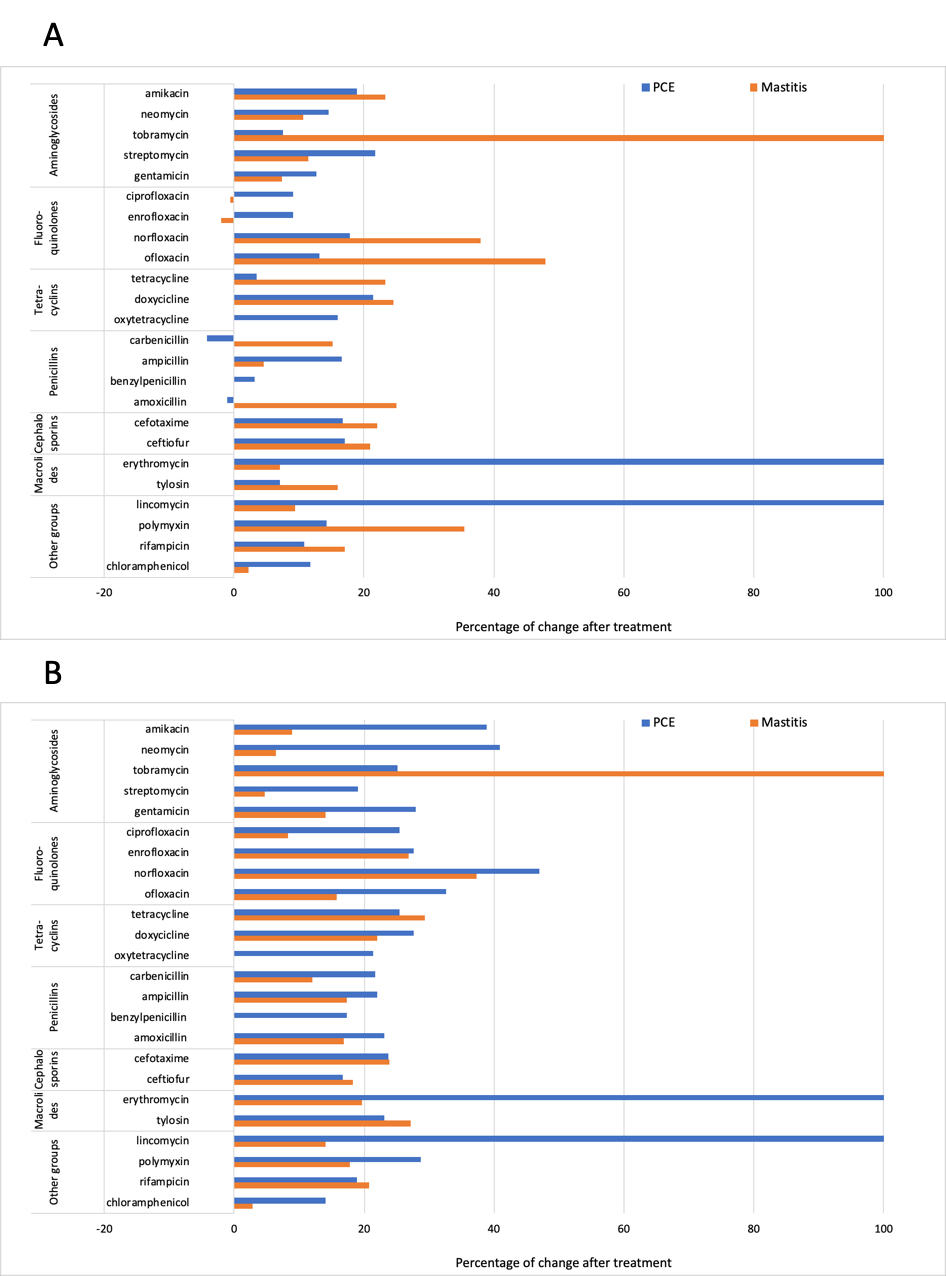


A comparison of percentage of antibiotic sensitivity change after Argovit-C treatment is shown. A) *E. coli* isolates without efflux effect. B) *E. coli* isolates with efflux effect. *E. coli* isolates were obtained after treatments for PCE (blue bars) and mastitis (orange bars), respectively.
