# Supplementary material for: AgNPs treatment reduces time recovery and increases bacterial sensitivity to antibiotics in cow´s purulent catarrhal endometritis. A translational study
Source: PLoS One. 2025 Oct 29;20(10):e0335305. doi: 10.1371/journal.pone.0335305 (PMC12571309; doi:10.1371/journal.pone.0335305)
Supplement: S1 Table — (DOCX) [file pone.0335305.s002.docx]

**Supplementary Table 1** – Primer sequences for detection of resistance genes

| Gene | Primers (5´to 3´) | References |
| --- | --- | --- |
| *blaDHA* | Fw - CCAGAATCACAATCGCCACC  Rv - TATCAGCAGTGGCA GCCGT | Guo Q. et al. (2012)^39^ |
| *blaGES* | Fw - TATGGGGATCTGCCCGATAGA  Rv - AAAGGCTCACCTTAGGCGAC | Conrad L.S. et al. (2020)^40^ |

**REFERENCES**

(39) Guo, Q.; Wang, P.; Ma, Y.; Yang, Y.; Ye, X.; Wang, M. Co-Production of SFO-1 and DHA-1 β-Lactamases and 16S RRNA Methylase ArmA in Clinical Isolates of Klebsiella Pneumoniae. *J. Antimicrob. Chemother.* **2012**, *67* (10), 2361–2366. https://doi.org/10.1093/JAC/DKS244.

(40) Schoch, C. L.; Ciufo, S.; Domrachev, M.; Hotton, C. L.; Kannan, S.; Khovanskaya, R.; Leipe, D.; McVeigh, R.; O’Neill, K.; Robbertse, B.; Sharma, S.; Soussov, V.; Sullivan, J. P.; Sun, L.; Turner, S.; Karsch-Mizrachi, I. NCBI Taxonomy: A Comprehensive Update on Curation, Resources and Tools. *Database (Oxford).* **2020**, *2020*. https://doi.org/10.1093/DATABASE/BAAA062.
