# Supplementary material for: AgNPs treatment reduces time recovery and increases bacterial sensitivity to antibiotics in cow´s purulent catarrhal endometritis. A translational study
Source: PLoS One. 2025 Oct 29;20(10):e0335305. doi: 10.1371/journal.pone.0335305 (PMC12571309; doi:10.1371/journal.pone.0335305)
Supplement: S2 Table — (DOCX) [file pone.0335305.s003.docx]

**Supplementary Table 2.** Antibiotic sensitivity test of *E. coli* isolates from cows’ PCE before and after treatment with Enrocide (in millimeters of growth inhibition).

| Groups | Drug | With an efflux effect | | | | %** |  | Without efflux effect | | | %** |
| --- | --- | --- | --- | --- | --- | --- | --- | --- | --- | --- | --- |
|  |  | n* | before treatment, mm | n* | after treatment, mm |  | n* | before treatment, mm | n* | after treatment, mm |  |
| Amino-glycosides | amikacin | 11 | 15,2+0,1 | 18 | 12,3+0,1 | -19 | 12 | 14,2+0,01 | 9 | 11,6+0,1 | -18,3 |
|  | neomycin | 9 | 14,7+0,1 | 19 | 12,8+0,1 | -12,9 | 17 | 14,4+0,04 | 10 | 12,1+0,1 | -15,9 |
|  | tobramycin | 15 | 16,5+0,1 | 22 | 14,4+0,1 | -12,7 | 14 | 15,7+0,01 | 11 | 12,1+0,04 | -22,9 |
|  | streptomycin | 8 | 18,3+0,1 | 18 | 15,1+0,1 | -17,4 | 19 | 15,9+0,1 | 16 | 13,8+0,03 | -13,2 |
|  | gentamicin | 12 | 16,2+0,1 | 17 | 14,3+0,1 | -11,7 | 11 | 15,4+0,03 | 10 | 12,7+0,1 | -17,5 |
| Fluoro-quinolones | ciprofloxacin | 10 | 17,1+0,1 | 17 | 17,9+0,1 | 4,6 | 16 | 16,8+0,1 | 11 | 17,5+0,1 | 4,1 |
|  | enrofloxacin | 12 | 20,7+0,1 | 16 | 16,3+0,1 | -21,2 | 14 | 16,1+0,1 | 9 | 14,1+0,1 | -12,4 |
|  | norfloxacin | 14 | 16,4+0,1 | 20 | 15,3+0,1 | -6,7 | 12 | 15,2+0,1 | 13 | 11,7+0,1 | -23 |
|  | ofloxacin | 10 | 12,3+0,1 | 16 | 12,9+0,1 | 4,8 | 11 | 11,8+0,1 | 8 | 11,2+0,1 | -5.00 |
| Tetracyclines | tetracycline | 15 | 16,8+0,1 | 21 | 15,0+0,1 | -10,7 | 18 | 15,9+0,1 | 10 | 12,7+0,1 | -20,1 |
|  | doxycicline | 11 | 17,5+0,1 | 19 | 14,7+0,1 | -16 | 16 | 16,7+0,1 | 14 | 13,5+0,1 | -19,1 |
|  | Oxytetracycline | 16 | 16,8+0,1 | 27 | 14,5+0,01 | -13,7 | 32 | 15,1+0,1 | 19 | 12,2+0,06 | -19,2 |
| Penicillins | carbenicillin | 12 | 16,1+0,1 | 17 | 11,9+0,1 | -26 | 20 | 13,2+0,1 | 15 | 11,5+0,01 | -12,8 |
|  | ampicillin | 9 | 16,5+0,1 | 14 | 13,2+0,04 | -20 | 16 | 16,1+0,03 | 12 | 11,7+0,1 | -27,3 |
|  | Benzylpenicillin | 12 | 11,8+0,1 | 16 | 10,5+0,01 | -11 | 14 | 11,4+0,1 | 11 | 10,3+0,1 | -9,6 |
|  | amoxicillin | 16 | 16,5+0,1 | 18 | 11,7+0,07 | -29 | 11 | 15,2+0,1 | 18 | 12,7+0,04 | -16,4 |
| Cephalos-porins | cefotaxime | 12 | 17,4+0,1 | 19 | 14,6+0,03 | -16 | 14 | 15,9+0,1 | 11 | 13,2+0,1 | -16,9 |
|  | ceftiofur | 7 | 17,6+0,1 | 21 | 13,1+0,1 | -25,5 | 16 | 16,1+0,1 | 14 | 13,5+0,06 | -16,1 |
| Macrolides | erythromycin | **-** | **LoS** | **-** | **LoS** | **-** | **-** | **LoS** | **-** | **LoS** | **-** |
|  | tylosin | 10 | 15,2+0,1 | 16 | 13,8+0,1 | -14,9 | 16 | 14,7+0,1 | 11 | 11,8+0,1 | -19,7 |
| Other groups | lincomycin | **-** | **LoS** | **-** | **LoS** | **-** | **-** | **LoS** | **-** | **LoS** | **-** |
|  | polymyxin | 10 | 16,2+0,1 | 17 | 12,4+0,05 | -23,4 | 16 | 12,8+0,1 | 12 | 11,4+0,03 | -10,9 |
|  | rifampicin | 9 | 16,1+0,1 | 18 | 14,7+0,07 | -8,6 | 14 | 14,1+0,06 | 10 | 12,4+0,04 | -12 |
|  | chloramphenicol | 8 | 16,9+0,1 | 21 | 15,4+0,1 | -8,8 | 13 | 14,3+0,1 | 7 | 11,1+0,1 | -22,3 |

n* - number of isolates, %** - percentage of antibiotic activity change after treatments, LoS - lack of sensitivity (Resistant bacteria).
