# Supplementary material for: AgNPs treatment reduces time recovery and increases bacterial sensitivity to antibiotics in cow´s purulent catarrhal endometritis. A translational study
Source: PLoS One. 2025 Oct 29;20(10):e0335305. doi: 10.1371/journal.pone.0335305 (PMC12571309; doi:10.1371/journal.pone.0335305)
Supplement: S3 Table — (DOCX) [file pone.0335305.s004.docx]

**Supplementary Table 3.** Antibiotic sensitivity test of *E. coli* isolates from cow´s PCE before and after treatment with Argovit-C (in millimeters of growth inhibition)

| Groups | Drug | With an efflux effect | | | | %** | Without efflux еeffect | | | | %** |
| --- | --- | --- | --- | --- | --- | --- | --- | --- | --- | --- | --- |
|  |  | n* | before treatment, mm | n* | after treatment, mm |  | n* | before treatment, mm | n* | after treatment, mm |  |
| Amino-glycosides | amikacin | 18 | 15,3+0,1 | 10 | 18,2+0,1 | 18,9 | 12 | 13,9+0,1 | 16 | 19,3+0,1 | 38,8 |
|  | neomycin | 12 | 15,1+0,1 | 6 | 17,3+0,1 | 14,5 | 11 | 13,2+0,04 | 15 | 18,6+0,1 | 40,9 |
|  | tobramycin | 12 | 16,9+0,1 | 5 | 18,2+0,1 | 7,6 | 14 | 14,7+0,01 | 18 | 18,4+0,06 | 25,1 |
|  | streptomycin | 16 | 17,5+0,1 | 10 | 21,3+0,1 | 21,7 | 18 | 15,7+0,03 | 20 | 18,7+0,02 | 19,1 |
|  | gentamicin | 19 | 17,3+0,1 | 5 | 19,5+0,1 | 12,7 | 16 | 15,7+0,06 | 14 | 20,1+0,1 | 28 |
| Fluoro-quinolones | ciprofloxacin | 12 | 17,5+0,1 | 7 | 19,1+0,1 | 9,1 | 18 | 16,5+0,1 | 20 | 20,7+0,1 | 25,4 |
|  | enrofloxacin | 18 | 20,7+0,1 | 8 | 22,6+0,1 | 9,1 | 14 | 15,9+0,1 | 18 | 20,3+0,1 | 27,6 |
|  | norfloxacin | 25 | 14,5+0,1 | 15 | 17,1+0,1 | 17,9 | 12 | 13,2+0,1 | 16 | 19,4+0,1 | 46,9 |
|  | ofloxacin | 18 | 17,4+0,1 | 7 | 19,7+0,1 | 13,2 | 14 | 13,8+0,1 | 16 | 18,3+0,1 | 32,6 |
| Tetracyclines | tetracycline | 14 | 17,2+0,1 | 11 | 17,8+0,1 | 3,4 | 12 | 14,5+0,1 | 16 | 18,2+0,1 | 25,5 |
|  | doxycicline | 17 | 16,8+0,1 | 6 | 20,4+0,1 | 21,4 | 18 | 15,4+0,1 | 21 | 19,6+0,1 | 27,2 |
|  | Oxytetracycline | 32 | 16,9+0,1 | 12 | 19,6+0,1 | 15,9 | 21 | 14,9+0,1 | 36 | 18,1+0,01 | 21,4 |
| Penicillins | carbenicillin | 15 | 14,5+0,1 | 11 | 13,9+0,1 | -4,1 | 14 | 15,2+0,1 | 24 | 18,5+0,1 | 21,7 |
|  | ampicillin | 17 | 16,9+0,1 | 11 | 19,7+0,1 | 16,5 | 11 | 15,4+0,04 | 14 | 18,8+0,02 | 22 |
|  | Benzylpenicillin | 19 | 12,3+0,1 | 14 | 12,7+0,1 | 3,2 | 9 | 10,3+0,1 | 12 | 12,1+0,1 | 17,4 |
|  | amoxicillin | 21 | 16,9+0,1 | 12 | 16,7+0,1 | -1,1 | 7 | 14,7+0,1 | 10 | 18,1+0,1 | 23,1 |
| Cephalos-porins | cefotaxime | 17 | 16,7+0,1 | 6 | 19,5+0,1 | 16,7 | 11 | 15,9+0,05 | 16 | 19,7+0,1 | 23,8 |
|  | ceftiofur | 12 | 16,9+0,1 | 9 | 19,8+0,1 | 17,1 | 9 | 16,1+0,1 | 12 | 18,8+0,06 | 16,7 |
| Macrolides | erythromycin | **-** | **LoS** | 2 | 12,1+0,1 | 100 | **-** | **LoS** | 4 | 12,4+0,1 | 100 |
|  | tylosin | 14 | 16,9+0,1 | 8 | 18,1+0,1 | 7,1 | 7 | 15,1+0,1 | 12 | 18,6+0,1 | 23,1 |
| Other groups | lincomycin | **-** | **LoS** | 2 | 11,4+0,1 | 100 | **-** | **LoS** | 4 | 11,7+0,1 | 100 |
|  | polymyxin | 12 | 15,3+0,1 | 9 | 17,5+0,03 | 14,3 | 11 | 13,9 +0,1 | 18 | 17,9+0,04 | 28,7 |
|  | rifampicin | 17 | 15,7+0,1 | 11 | 17,4+0,02 | 10,8 | 8 | 14,8+0,04 | 13 | 17,6+0,1 | 18,9 |
|  | chloramphenicol | 18 | 16,2+0,1 | 13 | 18,1+0,1 | 11,7 | 7 | 15,7+0,1 | 11 | 17,9+0,1 | 14 |

n* - number of isolates, %** - percentage of antibiotic activity change after treatments, LoS - lack of sensitivity (Resistant bacteria).
