# Supplementary material for: AgNPs treatment reduces time recovery and increases bacterial sensitivity to antibiotics in cow´s purulent catarrhal endometritis. A translational study
Source: PLoS One. 2025 Oct 29;20(10):e0335305. doi: 10.1371/journal.pone.0335305 (PMC12571309; doi:10.1371/journal.pone.0335305)
Supplement: S4 Table — (DOCX) [file pone.0335305.s005.docx]

**Supplementary Table 4.** Comparison of antibiotic sensitivity change of *E. coli* isolates from Purulent Catharral Endometritis (PCE) and mastitis after Argovit-C treatments.

*Mastitis data was obtained from Nefedova et al., 2023.
