# Supplementary material for: AgNPs treatment reduces time recovery and increases bacterial sensitivity to antibiotics in cow´s purulent catarrhal endometritis. A translational study
Source: PLoS One. 2025 Oct 29;20(10):e0335305. doi: 10.1371/journal.pone.0335305 (PMC12571309; doi:10.1371/journal.pone.0335305)
Supplement: S5 Table — (DOCX) [file pone.0335305.s006.docx]

**Supplementary Table 5.** Average of antibiotic sensitivity change of *E. coli* isolates from PCE and mastitis after Argovit-C treatments.

| Antibiotic group | With an efflux effect  Average of change | | Without an efflux effect  Average of change | |
| --- | --- | --- | --- | --- |
|  | PCE | Mastitis | PCE | Mastitis |
| Aminoglycosides | 15.1 (5.5) | 30.6 (39.3) | 30.4 (9.3) | 26.8 (41.1) |
| Fluoroquinolones | 12.3 (4.2) | 20.8 (25.9) | 33.1 (9.7) | 22.0 (12.7) |
| Tetracyclins | 13.6 (9.2) | 24.0 (0.9) | 24.9 (3.2) | 25.7 (5.2) |
| Penicillins | 3.6 (9.1) | 14.9 (10.3) | 21.1 (2.5) | 15.4 (3.0) |
| Cephalosporins | 16.9 (0.3) | 21.6 (0.8) | 20.3 (5.0) | 21.1 (4.0) |
| Macrolides | 53.6 (65.7) | 11.5 (6.4) | 61.6 (54.4) | 23.4 (5.3) |

Average from % of change found for antibiotics of each group shown in Supplementary Table 4.

Standard deviation is shown in parenthesis.
